# Evidence for Inhibitory Effects of Flupirtine, a Centrally Acting Analgesic, on Delayed Rectifier K^+^ Currents in Motor Neuron-Like Cells

**DOI:** 10.1155/2012/148403

**Published:** 2012-07-22

**Authors:** Sheng-Nan Wu, Ming-Chun Hsu, Yu-Kai Liao, Fang-Tzu Wu, Yuh-Jyh Jong, Yi-Ching Lo

**Affiliations:** ^1^Department of Physiology, National Cheng Kung University Medical College, Tainan City 70101, Taiwan; ^2^Institute of Basic Medical Sciences, National Cheng Kung University Medical College, Tainan City 70101, Taiwan; ^3^Department of Psychology, National Cheng Kung University, Tainan City 70101, Taiwan; ^4^Department of Pediatrics, Kaohsiung Medical University Chung-Ho Memorial Hospital, Kaohsiung City, Taiwan; ^5^Department of Pharmacology, Kaohsiung Medical University, Kaohsiung City 80708, Taiwan

## Abstract

Flupirtine (Flu), a triaminopyridine derivative, is a centrally acting, non-opiate analgesic agent. In this study, effects of Flu on K^+^ currents were explored in two types of motor neuron-like cells. Cell exposure to Flu decreased the amplitude of delayed rectifier K^+^ current (*I*_K(DR)_) with a concomitant raise in current inactivation in NSC-34 neuronal cells. The dissociation constant for Flu-mediated increase of *I*_K(DR)_ inactivation rate was about 9.8 *μ* M. Neither linopirdine (10 *μ* M), NMDA (30 *μ* M), nor gabazine (10 *μ* M) reversed Flu-induced changes in *I*_K(DR)_ inactivation. Addition of Flu shifted the inactivation curve of *I*_K(DR)_ to a hyperpolarized potential. Cumulative inactivation for *I*_K(DR)_ was elevated in the presence of this compound. Flu increased the amplitude of M-type K^+^ current (*I*_K(M)_) and produced a leftward shift in the activation curve of *I*_K(M)_. In another neuronal cells (NG108-15), Flu reduced *I*_K(DR)_ amplitude and enhanced the inactivation rate of *I*_K(DR)_. The results suggest that Flu acts as an open-channel blocker of delayed-rectifier K^+^ channels in motor neurons. Flu-induced block of *I*_K(DR)_ is unlinked to binding to NMDA or GABA receptors and the effects of this agent on K^+^ channels are not limited to its action on M-type K^+^ channels.

## 1. Introduction

Flupirtine (Flu) belongs to a class of triaminopyridines and is a centrally acting, nonopioid analgesic agent with muscle relaxing properties. It has been used for a wide variety of neurological disorders involving neuronal overexcitability such as epilepsy and neuropathic pain [1]. In addition to its use in pain management, Flu was also reported to display muscle relaxant and anticonvulsant activity. Notably, this compound was previously demonstrated to be beneficial in treating the patients with human prion diseases [2, 3]. A recent study showed the ability of Flu to reduce the myotonic membrane hyperexcitability induced by a Cl^−^ channel blocker [4].

 Earlier studies have demonstrated that it might act as an antagonist of N-methyl-D-aspartate (NMDA) receptors [5, 6]. A recent report also showed the ability of Flu to facilitate the activity of GABA_A_ receptors accompanied by increased stimulation of K_V_7 channels [7]. More importantly, it is accepted that Flu can bind to and activate voltage-gated K^+^ (K_V_) channels subtypes 7.2–7.5 (K_V_7.2–7.5). The increased activity of K_V_7.2, 7.3 and 7.5 channels generates the so-called M-type K^+^ current (*I*_K(M)_) which is a slowly activating and deactivating current suppressed by stimulation of muscarinic receptors [8–10]. Mutations of the *KCNQ2* gene are notably involved in peripheral nerve hyperexcitability, a syndrome which is characterized by a spontaneous and continuous muscle overactivity [11]. However, whether Flu and its structurally related compounds can produce any effects on other type of K_V_ channels remains unclear.

 The NSC-34 cell is a hybridoma cell line derived from the fusion of neuroblastoma cells with mice spinal cord cells. These cells have attracted growing interest as a suitable model for the evaluation of effects of potential neuroprotective compounds against different insults including excitotoxins, mitochondrial toxin, and oxidants [12, 13]. Previous observations in our laboratory have shown the presence of Na^+^-activated K^+^ channels functionally expressed in these cells [14].

 In this study, we attempted to evaluate the possible effects of Flu and other related compounds on ion currents in NSC-34 neuroblastoma-spinal cord hybrid cells and NG108-15 neuronal cells. Of note, we found that in NSC-34 and NG108-15 neuronal cells, addition of Flu is capable of producing inhibitory actions on delayed-rectifier K^+^ current (*I*_K(DR)_) in a concentration- and state-dependent fashion. The elevation of *I*_K(DR)_ inactivation rate caused by this compound was further quantitatively characterized in this study. The major action of this compound on *I*_K(DR)_ is thought to be through an open-channel mechanism. Findings from this study indicate that the inhibition by Flu of *I*_K(DR)_ in these neurons is not associated with a mechanism linked to the binding of either NMDA or GABA receptors and that the effects of Flu on K^+^ channels are not limited to its modification of M-type K^+^ channels.

## 2. Materials and Methods

### 2.1. Drugs and Solutions

Flupirtine maleate (Flu; ethyl{2-amino-6-[(4-fluorobenzyl)amino]pyridine-3-yl}carbamate; Katadolon), linopirdine, meclofenamic acid, 4-aminopyridine, tetraethylammonium chloride (TEA), and tetrodotoxin were obtained from Sigma-Aldrich (St. Louis, MO, USA). Gabazine and *N*-methyl-D-aspartic acid (NMDA) was from Tocris Cookson, Ltd. (Bristol, UK). Agitoxin-2, apamin, iberiotoxin, and margatoxin were obtained from Alomone Labs (Jerusalem, Israel). All culture media, fetal bovine serum, L-glutamine, trypsin/EDTA, and penicillin-streptomycin were obtained from Invitrogen (Carlsbad, CA, USA). All other chemicals were obtained from regular commercial chemicals and of reagent grade.

 The composition of the bath solution (i.e., normal Tyrode's solution) was 136.5 mM NaCl, 5.4 mM KCl, 1.8 mM CaCl_2_, 0.53 mM MgCl_2_, 5.5 mM glucose, and 5.5 mM HEPES-NaOH buffer, pH 7.4. To record *I*_K(DR)_ or *I*_K(M)_, the recording pipettes were backfilled with a solution consisting of 130 mM K-aspartate, 20 mM KCl, 1 mM KH_2_PO_4_, 1 mM MgCl_2_, 3 mM Na_2_ATP, 0.1 mM Na_2_GTP, 0.1 mM EGTA, and 5 mM HEPES-KOH buffer, pH 7.2.

### 2.2. Cell Preparations

 NSC-34 neuronal cells were originally produced by fusion of motor neuron-enriched, embryonic mouse spinal cords with mouse neuroblastoma [13, 14]. They were routinely maintained in 1 : 1 mixture of DMEM and Ham's F12 medium supplemented with 10% (v/v) fetal bovine serum (FBS) and 1% penicillin-streptomycin. Cultures were incubated at 37°C in a humidified environment of 5% CO_2_/95% air. The medium was often replenished every 2-3 days for removal of nonadhering cells. To slow cell proliferation and enhance their maturation towards a differentiated state, before confluence, cells were grown in 1 : 1 DMEM plus Ham's F12 medium supplemented with 1% FBS. NG108-15 mouse neuroblastoma x rat glioma hybrid cells, obtained from the European Collection of Cell Cultures (ECACC-88112302; Wiltshire, UK), were grown in monolayer cultures at a density of 10^6^/mL in plastic disks containing DMEM supplemented with 100 *μ*M hypoxanthine, 1 *μ*M aminopterin, 16 *μ*M thymidine, and 5% (v/v) FBS as the culture medium [15]. Electrophysiological experiments were performed 5 or 6 days after cells had been cultured (60–80% confluence). To observe neurite growth in these cells, a Nikon Eclipse Ti-E inverted microscope (Li Trading Co., Taipei, Taiwan) equipped with a five-megapixel cooled digital camera was commonly used. The camera was connected to a personal computer controlled by NIS-Element BR3.0 software (Nikon; Kanagawa, Japan).

### 2.3. RNA Isolation and Reverse Transcriptase-Polymerase Chain Reaction (RT-PCR)

 To detect the expression of K_V_3.1 mRNA in NSC-34 cells, a semiquantitative RT-PCR assay was performed. Total RNA samples were extracted from NSC-34 cells with TRIzol reagent (Invitrogen) and reverse-transcribed into complementary DNA using Superscript II reverse-transciptase (Invitrogen). The sequences of oligonucleotide primers used for K_V_3.1 (NM_008421) were 5′-CGTGCCGACGAGTTCTTCT-3′ and 5′-GGTCATCTCCAGCTCGTCCT-3′. Amplification of K_V_3.1 was made using PCR SuperMix from Invitrogen under the following conditions: 35 cycles composed of 30 sec denaturation at 95°C, 30 sec primer annealing at 62°C, 1 min extension at 72°C, and followed by 72°C for the final extension for 2 min. PCR products were analyzed on 1.5% (v/v) agarose gel containing ethidium bromide and then visualized under ultraviolet light. Optical densities of DNA bands were scanned and quantified by AlphaImager 2200 (ProteinSimple; Santa Clara, CA, USA).

### 2.4. Electrophysiological Measurements

NG108-15 or NSC-34 cells used for electrophysiological experiments were dissociated and an aliquot of cell suspension was transferred to a recording chamber mounted on the stage of an inverted fluorescent microscope (CKX-41; Olympus, Tokyo, Japan). Cells were bathed at room temperature (20–25°C) in normal Tyrode's solution containing 1.8 mM CaCl_2_. Patch pipettes were made from Kimax-51 glass capillaries (Kimble; Vineland, NJ, USA) using a PP-830 electrode puller (Narishige, Tokyo, Japan) or a P-97 Flaming/Brown micropipette puller (Sutter; Novato, CA, USA), and their tips were fire-polished with an MF-83 microforge (Narishige). The pipettes used had a resistance of 3–5 MΩ when immersed in different solutions as described above. Patch-clamp recordings were made in cell-attached or whole-cell configurations using an RK-400 (Bio-Logic, Claix, France) or an Axopatch 200B patch-clamp amplifier (Molecular Devices; Sunnyvale, CA, USA) [15, 16].

### 2.5. Data Analyses

The inhibitory effect of Flu on *I*_K(DR)_ in NSC-34 cells can be explained by a state-dependent blocker that binds to the open state of the channel according to a minimal kinetic scheme [16, 17]:

(1) C⇄αβO⇄kOBkBOO•B,

where *α* and *β* are the voltage-dependent rate constants for the opening and closing of the delayed-rectifier K^+^ (K_DR_) channel; *k*_OB_ and *k*_BO_, those for blocking and unblocking by Flu (i.e., the on- and off-rate constants for Flu) and [B] is the blocker (i.e., Flu) concentration. C, O, and O • B represent the closed, open, and open-blocked states, respectively.

Specifically, the blocking and unblocking rate constants, *k*_OB_ and *k*_BO_, were determined from the inactivation time constants (*τ*_inact_) for *I*_K(DR)_ evoked by the depolarizing pulses. Those values were estimated using the relation: 1/*τ*_inact_ = *k*_OB_ × [B] + *k*_BO_, where *k*_OB_ and *k*_BO_, respectively, result from the slope and the *y*-axis intercept at [B] = 0 of the linear regression interpolating the reciprocal time constants (i.e., 1/*τ*_inact_) versus different Flu concentrations.

The steady-state inactivation curve of *I*_K(DR)_ with or without addition of Flu was plotted against the conditioning pulses and fitted to the Boltzmann equation:

(2) I=Imax⁡1+exp⁡((V−V1/2)/k)  ,

where *V* is the conditioning potential in mV, *V*_1/2_ is the membrane potential for half-maximal inactivation, and k is the slope factor of inactivation curve for *I*_K(DR)_.

The relationships between the membrane potentials and the *I*_K(M)_ conductance obtained before and after the addition of Flu (3 *μ*M) were fitted with a Boltzmann function of the following form:

(3) GGmax⁡=11+exp⁡[−(V−V1/2)qF/RT],

where *G*_max⁡_ is the maximal conductance of *I*_K(M)_, *V*_1/2_ is the voltage at which there is half-maximal activation, *q* is the apparent gating charge, *F* is Faraday's constant, *R* is the universal gas constant, and *T* is the absolute temperature. Curve-fitting to data sets presented here was commonly performed using either Microsoft Excel (Redmond, WA, USA) or Origin 8.0 (OriginLab, Northampton, MA, USA).

Values are provided as means ± SEM with samples sizes (*n*) indicating the number of cells from which the data were taken. The paired or unpaired Student's  *t*-test and one-way analysis of variance (ANOVA) with the least significant different method for multiple comparisons were used for the statistical evaluations of differences among means. Nonparametric Kruskal-Wallis test was then used if the assumption of normality underlying ANOVA was violated. Statistical significance was determined at a *P*-value of <0.05.

## 3. Results

### 3.1. Effect of Flu on Delayed Rectifier K^+^ Current (*I*_K(DR)_) in Motor Neuronal NSC-34 Cells

**Figure 1:**
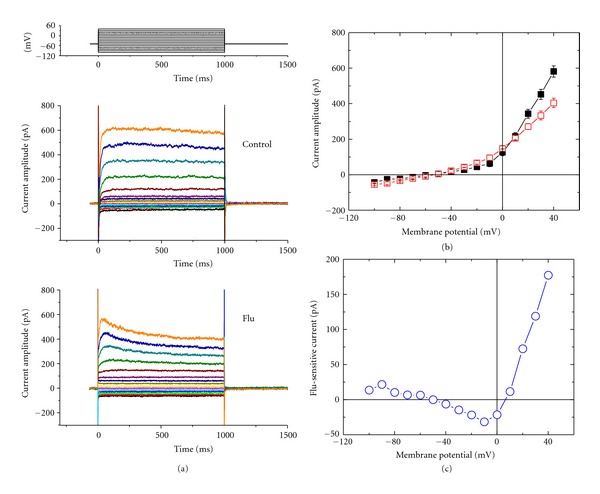
Effects of Flu on *I*_K(DR)_ in NSC-34 neuronal cells. Cells were bathed in Ca^2+^-free Tyrode's solution containing tetrodotoxin (1 *μ*M) and CdCl_2_ (0.5 mM). (a) Superimposed current traces in the absence (upper) and presence (lower) of 3 *μ*M Flu. The cell examined was held at −50 mV and various potentials ranging from −100 to +40 mV in 10-mV increments, as shown in the uppermost part of (a), were applied. (b) Averaged  *I*-*V*  relations for K^+^ currents measured at the end of each voltage pulse in the absence (■) and presence (□) of 3 *μ*M Flu (mean ± SEM; *n* = 9–13 for each point). (c) The  *I*-*V*  relationship of Flu-sensitive current (i.e., the difference of averaged K^+^ currents between the absence and presence of 3 *μ*M Flu).

 In this study, the whole-cell configuration of the patch-clamp technique was used to evaluate effects of Flu on ion current in NSC-34 cells. To record K^+^ outward currents, cells were bathed in Ca^2+^-free Tyrode's solution which contained tetrodotoxin (1 *μ*M) and CdCl_2_ (0.5 mM). When the cell was held at −50 mV, various voltage pulses ranging from −100 to +40 mV in 10-mV increments were applied with a duration of 1 sec, a family of K^+^ currents was elicited (Figure 1). These outward currents which were subject to block by tetraethylammonium chloride or 4-aminopyridine, but not by iberiotoxin or apamin, was thus identified as *I*_K(DR)_ [16, 17]. Iberiotoxin and apamin are blockers of the large- and small-conductance Ca^2+^-activated K^+^ channels, respectively. When cells were exposed to Flu (3 *μ*M), the *I*_K(DR)_ amplitude was reduced together with increased current inactivation. For example, when cells were depolarized from −50 to +40 mV, Flu at a concentration of 3 *μ*M significantly decreased the current amplitude by 30 ± 2% from 581 ± 32 pA to 404 ± 26 pA (*n* = 11). After washout of drug, current amplitude was partially returned to the control. The *I*-*V* relationships of *I*_K(DR)_ with or without addition of Flu are illustrated (Figure 1(b)). It was also noted that the current amplitudes at the voltages between −50 and +10 mV were increased in the presence of Flu. Figure 1(c) depicts an  *I*-*V*  relationship of Flu-sensitive current which could not result from the contamination with either Na^+^ or Ca^2+^ currents, because bathing solution in this study contained tetrodotoxin and CdCl_2_.

### 3.2. Kinetic Studies of Flu-Induced Block of *I*_K(DR)_

**Figure 2:**
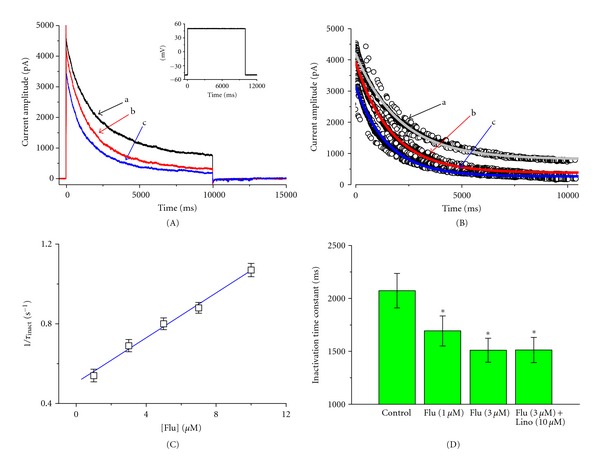
Evaluation of the kinetics of Flu-induced block of *I*_K(DR)_ in NSC-34 neuronal cells. In (A), the time courses of current inactivation obtained in the absence (a) and presence of 1 *μ*M (b) and 3 *μ*M (c) Flu were well fitted by a single exponential shown in (B). Inset in (A) indicates the voltage protocol used. In (C), the kinetics of Flu-induced block of *I*_K(DR)_ in NSC-34 cells was evaluated. The reciprocal of inactivation time constant of *I*_K(DR)_ (1/*τ*_inact_) obtained by exponential fit to the trajectory of *I*_K(DR)_ decay was plotted against the Flu concentration. Data points were fitted by a linear regression shown in blue line, indicating that Flu-induced blocking occurs with a molecularity of 1. Blocking (*k*_OB_) and unblocking (*k*_BO_) rate constants, given by the slope and the *y*-axis intercept of the interpolated line, were 0.0564 sec^−1^ *μ*M^−1^ and 0.504 sec^−1^, respectively. (D) Summary of the data showing the effect of Flu and linopirdine (Lino; 10 *μ*M) on the inactivation time constants (*τ*_inact_) of *I*_K(DR)_ elicited by a 10-sec depolarizing pulse from −50 to +50 mV (mean ± SEM; *n* = 8–10 for each point). In the experiments with Flu plus linopirdine, linopirdine (10 *μ*M) was applied after the addition of Flu (3 *μ*M). *Significantly different from control.

 Because *I*_K(DR)_ observed in the presence of Flu tends to exhibit a pronounced peak followed by an exponential decay to a steady-state level, it is necessary to evaluate the kinetics of Flu-induced block of *I*_K(DR)_ in NSC-34 cells. To provide more evidence for Flu-induced blocking of *I*_K(DR)_, the time constants for *I*_K(DR)_ inactivation in NSC-34 cells were further analyzed. The time courses of current inactivation in the absence and presence of different Flu concentrations were fitted by a single-exponential function. The concentration dependence of *I*_K(DR)_ elicited by sustained depolarizations with or without addition of Flu is illustrated in Figure 2. The results showed that the effects of this compound were concentration-dependent increase in the rate of current inactivation accompanied by a decrease in the residual, steady-state current. For example, when cells were depolarized from −50 to +50 mV with a duration of 10 sec, the inactivation time constants of *I*_K(DR)_ during exposure to 1 and 3 *μ*M Flu were fitted by a single exponential with the values of 1687 ± 13 msec (*n* = 8) and 1509 ± 12 msec (*n* = 9), respectively.

 Based on the first-order blocking scheme described in Section 2, the relationship between 1/*τ*_inact_ and [B] became linear with a correlation coefficient of 0.96 (Figure 2(c)). The blocking and unblocking rate constants measured from eight to eleven different cells were calculated to be 0.0564 sec^−1^ *μ*M^−1^ and 0.504 sec^−1^, respectively. Thereafter, based on these values, a value of 8.9 *μ*M for the dissociation constant (K_*D*_ = *k*_BO_/*k*_OB_) could be calculated.

Previous studies have demonstrated that Flu could activate *I*_K(M)_ [9]. We also examined whether linopirdine, a blocker of *I*_K(M)_, had any effects on Flu-induced perturbations of *I*_K(DR)_ amplitude. As shown Figure 2(d), linopirdine at a concentration of 10 *μ*M had little or no effect on the inactivation time constant of *I*_K(DR)_ elicited by membrane depolarization. Therefore, Flu-induced elevation of *I*_K(DR)_ inactivation rate in NSC-34 cells did not result from the activation of *I*_K(M)_.

### 3.3. Lack of Effect of N-Methyl-D-Aspartate (NMDA) or Gabazine on Flu-Induced Changes in *I*_K(DR)_

**Figure 3:**
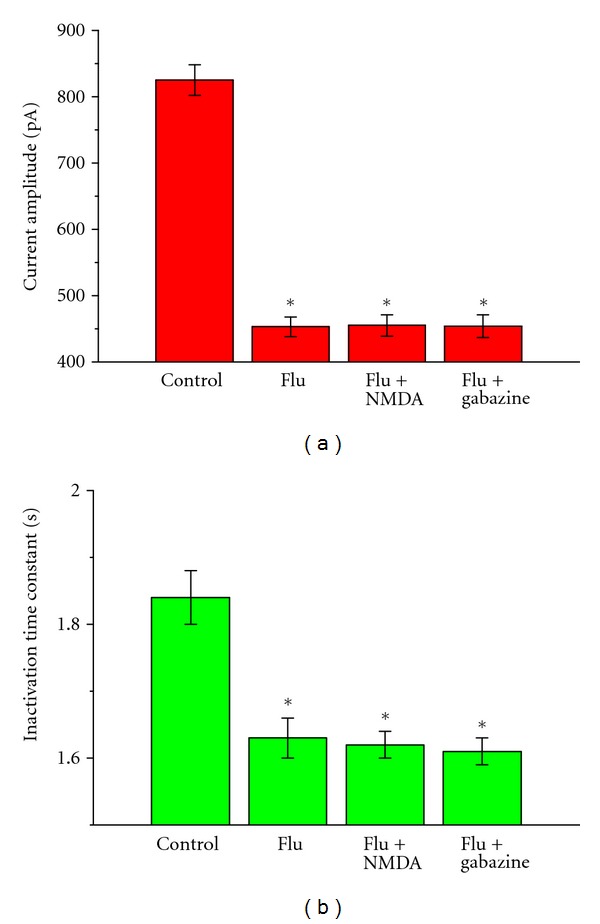
Lack of effect of NMDA or gabazine on Flu-induced effect on *I*_K(DR)_ in NSC-34 cells. In these experiments, each cell was depolarized from −50 to +50 mV with a duration of 10 sec. Current amplitude at the end of depolarizing pulse was measured and current inactivation time course was fitted by a single exponential. Bar graphs in (a) and (b) show the effects of Flu (3 *μ*M), Flu (3 *μ*M) plus NMDA (30 *μ*M), and Flu (3 *μ*M) plus gabazine (10 mM) on the peak amplitude (a) and inactivation time constant (b) of *I*_K(DR)_, respectively. In the experiments with Flu plus NMDA or gabazine, NMDA (30 *μ*M) or gabazine (10 *μ*M) was applied after the addition of Flu (3 *μ*M). Each bar represents the mean ± SEM (*n* = 8–12). *Significantly different from controls. Notably, subsequent application of NMDA or gabazine did not alter the amplitude and inactivation time course of *I*_K(DR)_.

 Several reports showed that Flu could interact with either glutamate NMDA or GABA receptors, thereby influencing the activity of ion channels in central neurons [5–7, 18]. We further evaluated whether the challenging of cells with NMDA, an NMDA agonist, or gabazine, a blocker of GABA_A_ receptor, can exert any effects on Flu-induced changes in *I*_K(DR)_ in these cells. The experiments were conducted with a 10-sec depolarizing pulse from a holding potential of −50 mV. Current amplitude at the end of each depolarizing pulse was measured and the inactivation time constant for *I*_K(DR)_ was analyzed. As shown in Figure 3, subsequent application of NMDA (30 *μ*M) or gabazine (10 *μ*M) had minimal effects on both current amplitude and inactivation elicited by long-lasting membrane depolarization. The observed effect of Flu on *I*_K(DR)_ inactivation is thus unlinked to the binding to NMDA or GABA receptors and most likely due to the drug acting on the channel itself.

### 3.4. Steady-State Inactivation of *I*_K(DR)_ Obtained with or without Addition of Flu

**Figure 4:**
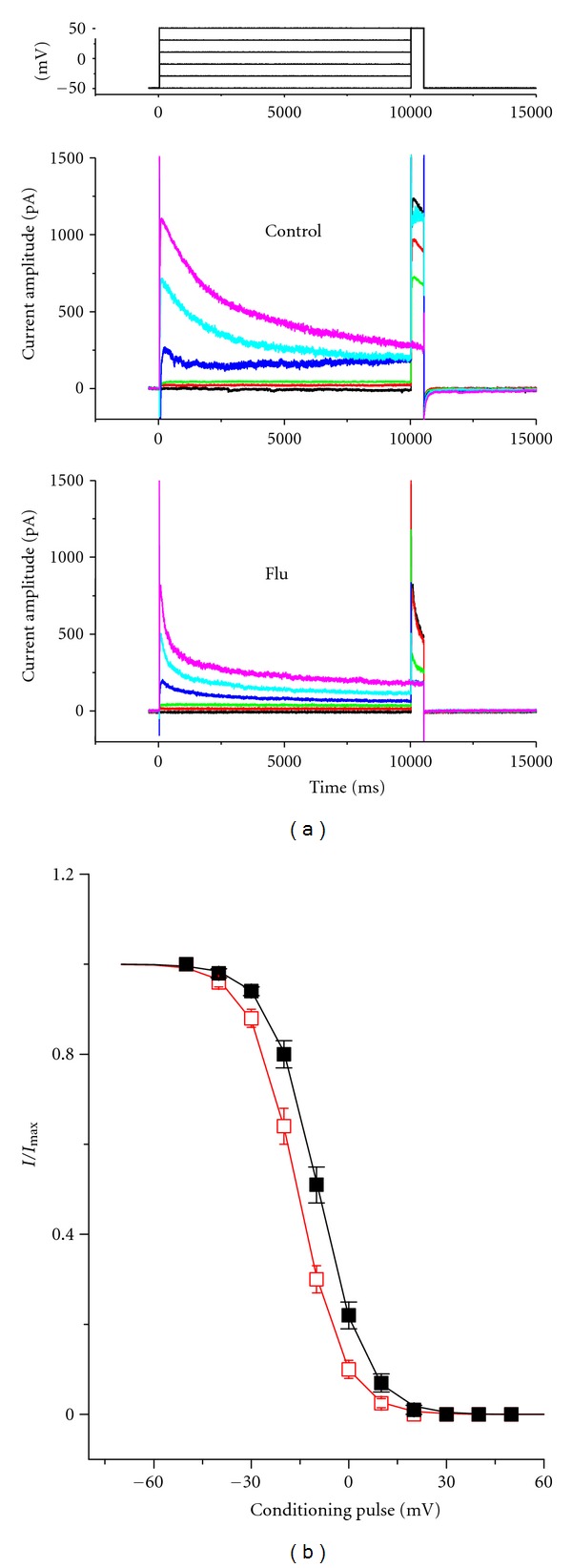
Effect of Flu on steady-state inactivation of *I*_K(DR)_ in NSC-34 cells. (a) Superimposed current traces obtained in the absence (upper) and presence (lower) of 3 *μ*M Flu. The conditioning voltage pulses with a duration of 10 sec to various membrane potentials. The voltage protocol used is shown in the uppermost part. (b) Steady-state inactivation of *I*_K(DR)_ obtained in the absence (■) and presence (□) of 3 *μ*M Flu (mean ± SEM; *n* = 8–11 for each point).

 To characterize the inhibitory effect of Flu on *I*_K(DR)_ in NSC-34 cells, we next investigated the voltage-dependence of its effect on *I*_K(DR)_ using a double-pulse protocol.  Figure 4 shows the steady-state inactivation curve of *I*_K(DR)_ in the absence and presence of Flu (3 *μ*M). In these experiments, a 10-sec conditioning pulse to different potentials preceded the test pulse to +50 mV from a holding potential of −50 mV. The relationship between the conditioning potentials and the normalized amplitudes of *I*_K(DR)_ were derived and constructed with a Boltzmann function, as described in Materials and Methods. In controls, voltage for half-maximal inactivation (*V*_1/2_) and corresponding slope factor (*k*) were −9.5 ± 0.4 mV and 7.4 ± 0.3 mV (*n* = 9), respectively, while in the presence of Flu (3 *μ*M), the values of *V*_1/2_ and *k* were −15.9 ± 0.6 mV and 7.2 ± 0.3 mV (*n* = 10), respectively. Therefore, addition of Flu slightly but significantly shifted the midpoint of the inactivation curve toward hyperpolarizing voltage by approximately 6.4 mV; however, no significant change in the slope factor was detected during exposure to Flu.

### 3.5. Flu-Induced Increase in Cumulative Inhibition of *I*_K(DR)_ Inactivation

**Figure 5:**
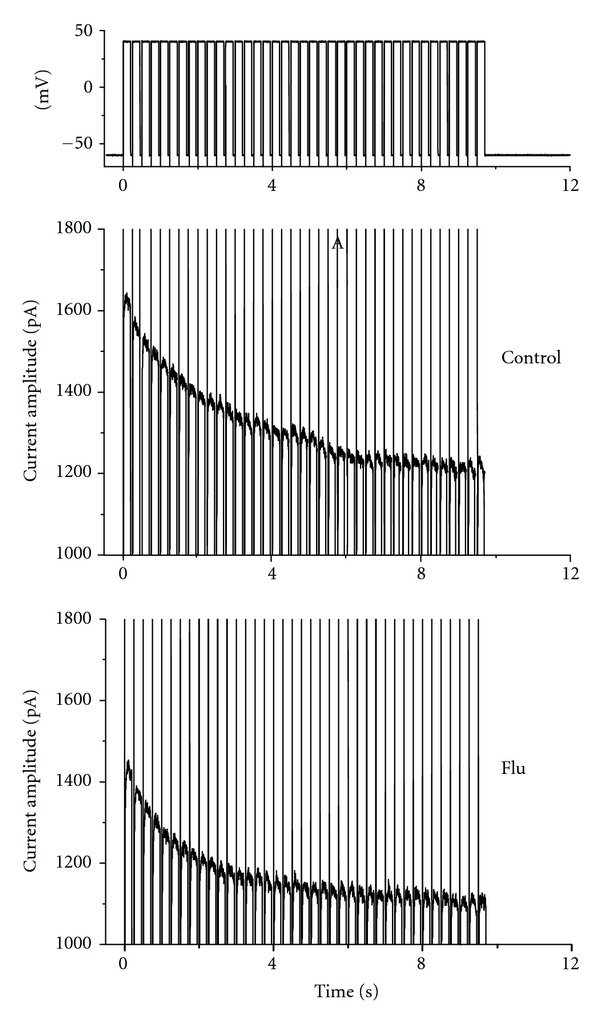
Excessive accumulative inactivation of *I*_K(DR)_ during repetitive stimuli in the absence (upper) and presence (lower) of 3 *μ*M Flu recorded from motor neuronal NSC-34 cells. Currents were obtained during a 20-sec repetitive depolarizations to +40 mV from a holding potential of −60 mV. The uppermost part indicates the voltage protocol used. Note that in addition to the inhibition of *I*_K(DR)_ amplitude, Flu increases the rate of excessive accumulative inactivation of *I*_K(DR)_ elicited by repetitive stimuli.

 In another set of experiments, we sought to determine whether Flu can alter the time course of *I*_K(DR)_ in response to repetitive stimuli in NSC-34 cells. Under control condition, a single 10-sec depolarizing step to +40 mV from a holding potential of −60 mV produced an exponential decline with a time constant of 4.09 ± 0.08 sec (*n* = 8). However, the time constant for 10-sec repetitive pulses to +40 mV, each of which lasted 20 msec with 10-msec interval at −60 mV between the depolarizing pulses, was significantly reduced to 3.51 ± 0.07 sec (*n* = 7). Additionally, as depicted in Figure 5, the results show a progressive increase in the decline of *I*_K(DR)_ in response to rapid depolarizing stimuli. When the cells were exposed to Flu, the value of time constant obtained during this train of short repetitive pulses was further reduced. Addition of 1 and 3 *μ*M Flu decreased the time constants to 2.54 ± 0.08 sec (*n* = 7) and 1.81 ± 0.07 sec (*n* = 6), respectively. However, subsequent application of linopirdine (10 *μ*M) did not alter the inactivation time constant for *I*_K(DR)_ in response to such repetitive stimuli (data not shown). Therefore, it needs to be noted that an excessive accumulative inactivation of *I*_*K*(DR)_ in the presence of Flu can be observed and not directly relevant to its stimulation of *I*_K(M)_.

### 3.6. Stimulatory Effect on *I*_K(M)_ in NSC-34 Cells

 Previous studies have demonstrated the presence of *I*_K(M)_ in NG108-15 neuronal cells [15, 19]. The experimental results shown in Figure 1 suggest that Flu can increase the *I*_K(DR)_ amplitude recorded at the voltages between −50 and +10 mV. We next investigated the effect of Flu on *I*_K(M)_ found in NSC-34 cells. When cells were hyperpolarized from −20 to −50 mV, the *I*_K(M)_ amplitudes in the presence of Flu were significantly greater than those in the control. Similarly, addition of meclofenamic acid (30 *μ*M), an activator of *KCNQ2*/Q3 channels [20], elevated the amplitude of *I*_K(M)_ (data not shown).

**Figure 6:**
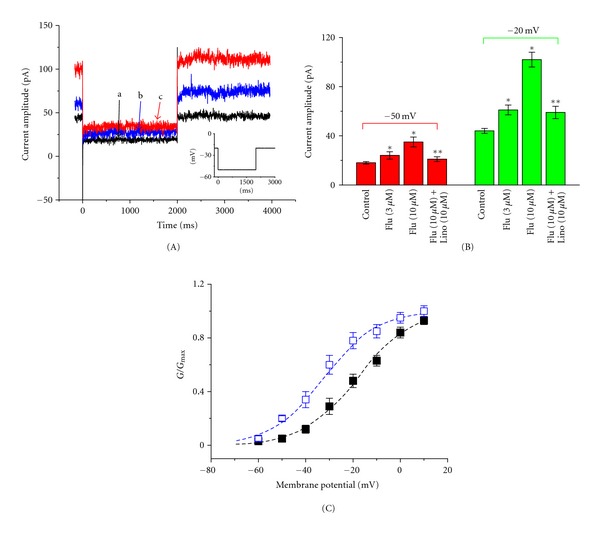
Stimulatory effect of Flu on M-type K^+^ current (*I*_K(M)_) in NSC-34 cells. In these experiments, cells were bathed in Ca^2+^-free Tyrode's solution and the pipette was filled with a K^+^-containing solution. (A) Current traces obtained in the absence (a) and presence of 1 and 3 *μ*M Flu. The inset indicates the voltage protocol used. (B) Summary of the data showing effects of Flu and linopirdine on *I*_K(M)_ measured at −50 mV (left) and −20 mV (right) in NSC-34 cells (mean ± SEM; *n* = 10–13 for each point). Flu: flupirtine; Lino: linopirdine. *Significantly different from controls. **Significantly different from Flu (3 *μ*M) alone group. (C) Voltage dependence of *I*_K(M)_ activation in the absence (■) and presence (□) of 3 *μ*M Flu (mean ± SEM; *n* = 7–10 for each point). The smooth dashed lines represent the best fits to the Boltzmann equation described in Section 2. Notably, addition of Flu can shift the activation curve of *I*_K(M)_ to the hyperpolarizing voltage with no change in the gating charge of this current.

Figure 6(c) shows the activation curve of *I*_K(M)_ in the absence and presence of Flu (3 *μ*M). The plot of relative *I*_K(M)_ conductance as a function of membrane potential was fitted with a Boltzmann function as described under Materials and Methods. In controls, *V*_1/2_ = −18.3 ± 0.6 mV and *q* = 2.25 ± 0.08 e (*n* = 8), whereas in the presence of Flu (3 *μ*M), *V*_1/2_ = −32.6 ± 1.1 mV and *q* = 2.31 ± 0.09 e (*n* = 7). The data showed that the activation curve was shifted along the voltage axis to more negative potentials by approximately 14 mV, as NSC-34 cells were exposed to Flu. In contrast, no significant change in the gating charge was demonstrated during exposure to Flu. These results thus indicate that Flu is capable of stimulating *I*_K(M)_ in a voltage-dependent fashion in NSC-34 cells.

### 3.7. Effect of Flu on *I*_K(DR)_ in NG108-15 Neuronal Cells

**Figure 7:**
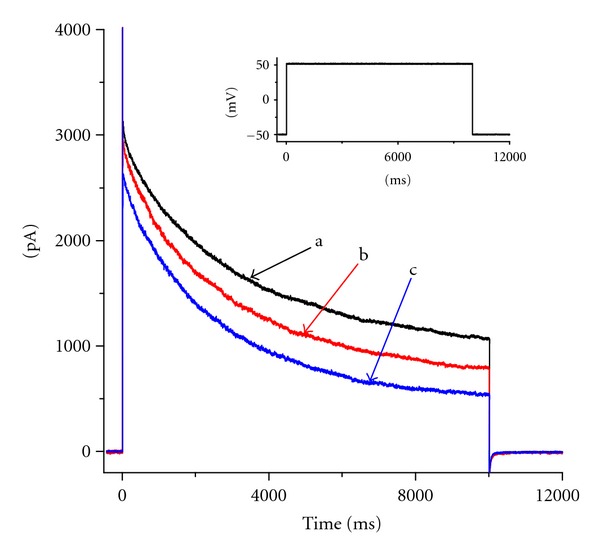
Inhibitory effect of Flu on *I*_K(DR)_ in NG108-15 neuronal cells. Cells were bathed in Ca^2+^-free Tyrode's solution and *I*_K(DR)_ was elicited by the depolarizing pulse from −50 to +50 mV with a duration of 10 sec. a: control; b: 1 *μ*M Flu; c: 3 *μ*M Flu. The inset indicates the voltage protocol used. Notably, addition of Flu increases the *I*_K(DR)_ inactivation rate along with the reduced current amplitude.

 Previous studies in our laboratory have demonstrated the presence of *I*_K(DR)_ (i.e., K_V_3.1-encoded current) in NG108-15 neuronal cells [15–17]. In a final set of experiments, to verify whether Flu-induced inhibition of *I*_K(DR)_ could also be observed in other types of neuronal cells, we examined the effects of this compound on NG108-15 neuronal cells. As shown in Figure 7, Flu was effective in suppressing *I*_K(DR)_ enriched in these cells. Besides that, it was able to raise the inactivation rate of *I*_K(DR)_ in response to a long-lasting depolarizing pulse. Similar to the results in NSC-34 cells, linopirdine at a concentration of 10 *μ*M was unable to alter the inactivation time constant of *I*_K(DR)_ decreased by Flu.

## 4. Discussion

This study demonstrated that in motor neuronal NSC-34 cells, Flu, which is a centrally acting, nonopioid analgesic, induced a time-, concentration-, and state-dependent decay of *I*_K(DR)_, although no change in activation kinetics of this current was found. These observations, along with a good description of *I*_K(DR)_ inactivation time course at different Flu concentrations with the simulation of minimal binding scheme, led us to propose that Flu may act as a state-dependent blocker for *I*_K(DR)_. Block by Flu of *I*_K(DR)_ is potentially important because it may have characteristics that render it significant from a pharmacological viewpoint.

 In our study, NSC-34 cell line was found to exhibit the activity of the slowly inactivating delayed rectifier K^+^ channels as described in NG108-15 neuronal cells [16, 17]. A notable characteristic for the majority of *I*_K(DR)_ in NSC-34 cells is that it activates rapidly and then inactivates slowly. The activation time constant ranges from 20 to 50 msec at the potentials between +30 and +70 mV; however, it tends to inactivate slowly from several hundreds to several thousands of milliseconds during sustained depolarization. The kinetics of K_V_3.1-encoded current thus fit well with these properties [16, 17, 21]. It is anticipated that K_V_3.1-encoded currents are the major molecular component of *I*_K(DR)_ in NSC-34 cells. However, because neither margatoxin (100 nM) nor agitoxin-2 (100 nM) produce any effects on *I*_K(DR)_ in NSC-34 cells (data not shown), there is most likely to be lack of functional expression of K_V_1 subunits in these cells. Margatoxin is a selective inhibitor of K_V_1.3 channel and agitoxin is a blocker to *Shaker* K^+^ channels.

 The observed effect of Flu on *I*_K(DR)_ in NSC-34 cells clearly did not involve the suppression of Ca^2+^-dependent K^+^ channels. The recordings of *I*_K(DR)_ presented herein were commonly conducted in a Ca^2+^-free Tyrode's solution containing CdCl_2_ (0.5 mM). Moreover, neither peak amplitude nor inactivation kinetics of *I*_K(DR)_ in NSC-34 cells remained altered during exposure to iberiotoxin or apamin. Iberiotoxin and apamin are known to block the large- and small-conductance Ca^2+^-activated K^+^ channels, respectively. However, the block of *I*_K(DR)_ by Flu could be responsible for the widening of neuronal action potentials [16, 21].

 In this study, NMDA, an NMDA receptor agonist, did not produce any effects on Flu-induced reduction of *I*_K(DR)_; therefore, the inhibitory effect of Flu on *I*_K(DR)_ described here would not be linked to an interaction with NMDA receptors. Additionally, gabazine (10 *μ*M), a blocker of GABA_A_ receptor, also did not exert any effect on Flu-induced reduction of *I*_K(DR)_ inactivation and amplitude. Therefore, Flu-induced perturbations in *I*_K(DR)_ kinetics is not because of the ability to bind to GABA_A_ receptors.

 The K_*D*_ value of Flu required for the increase of *I*_K(DR)_ inactivation rate was 8.9 *μ*M. Linopirdine did not reverse Flu-induced increase of *I*_K(DR)_ inactivation rate. After a single oral application of 200 mg Flu or the therapeutic daily dose of 600 mg Flu, the peak plasma concentration of 2.4 *μ*g/mL (6 *μ*M) can be reached [1, 9, 22]. It is possible that the Flu concentration used to alter the kinetics of *I*_K(DR)_ is compatible with clinically relevant concentrations.

 We also demonstrated the presence of *I*_K(M)_ in NSC-34 cells. Addition of Flu was noted to increase *I*_K(M)_ amplitude and shift the activation curve of this curve to more negative potentials, although no change in the gating charge was demonstrated. Because of lack of Flu on the gating charge in *I*_K(M)_, the voltage-sensor regions of neuronal KCNQ channels might not be altered by this compound. Subsequent application of linopirdine can reverse Flu-stimulated *I*_K(M)_. Besides that, the effects of Flu and meclofenamic acid on the *I*_K(M)_ amplitude in NSC-34 cells were not additive (data not shown), suggesting that the two compounds exert their major effects on the same component. However, the ability of Flu to suppress *I*_K(DR)_ amplitude and to increase the inactivation time course of this current in neurons is necessarily noted with caution in relation to its use as an activator of neuronal KCNQ channels.

 The K_V_3.1-encoded channels were enriched in many central neurons including hippocampal pyramidal neurons, auditory neurons, and Purkinje cells [21]. The activity of these channels is recognized to participate in electrical behaviors of fast-spiking neurons [21, 23]. Addition of Flu reduced the peak amplitude of *I*_K(DR)_ as well as increased the inactivation time course of this current recorded from NSC-34 cells. The present observations would clearly initiate further studies to understand the Flu effects on electrical activity of motor neurons [4, 24, 25]. Whether Flu used in treating patients with Creutzfeld-Jakob disease [1–3, 26] relates to an inhibition of *I*_K(DR)_ needs further evaluations.

 In summary, both stimulation of *I*_K(M)_ and inhibition of *I*_K(DR)_ caused by Flu may synergistically act to diminish the firing activity of motor neurons if similar results occur *in vivo*. Our results are novel but are based on two different types of neuronal cells and require replication in central neurons from different regions. Nonetheless, in addition to its stimulatory effect on *I*_K(M)_, the slowly inactivating *I*_K(DR)_ presented herein may be a relevant target for the action of Flu, if these types of K^+^ currents perturbed by this agent are present in myelinated neurons *in vivo*.
